# Towards person-centered pain management in dementia: usability of a digital medical device in Portuguese residential care facilities

**DOI:** 10.3389/frhs.2026.1829082

**Published:** 2026-06-24

**Authors:** Isa Brito Félix, Margarida Fernandes, Jeffery David Hughes, Kreshnik Hoti, Mara Pereira Guerreiro

**Affiliations:** 1 Egas Moniz Center for Interdisciplinary Research (CiiEM), Egas Moniz School of Health & Science, Caparica, Almada, Portugal; 2 Egas Moniz School of Health & Science, Caparica, Almada, Portugal; 3 PainChek® Ltd, Sydney, NSW, Australia; 4 Curtin Medical School, Curtin University, Perth, WA, Australia; 5 Faculty of Medicine, University of Prishtina, Prishtina, Kosovo

**Keywords:** dementia, pain assessment, PainChek, residential care facility, simulation environment, usability evaluation

## Abstract

**Introduction:**

Pain is common in people living with dementia (PLWD), yet frequently remains unrecognized and undertreated. Traditional observational tools are limited by subjectivity and suboptimal psychometric properties. The PainChek® mobile application (app) is a medical device that enables objective assessment in non-verbal people, such as those with more advanced dementia, using artificial intelligence (AI)-driven recognition of facial muscle movements. This study evaluated the usability of the Portuguese (PT) version of the PainChek® app among formal caregivers in residential care.

**Methods:**

Usability was evaluated in a controlled environment, guided by ISO 9241-11:2018 and ISO 25062:2025. After training, 19 participants performed nine tasks using the PainChek®-PT app while undertaking concurrent think-aloud; performance was screen and audio-recorded and supplemented with observation notes. Participants completed the Post-Study System Usability Questionnaire (PSSUQ-PT). Metrics included effectiveness (success rates, errors), efficiency (task completion time, attempts), satisfaction, usability problems and likely defects. Descriptive statistics were performed and confidence intervals (CI) computed, using 95% CIs for success rates and task times. PSSUQ scores were interpreted using 99% CIs against normative datasets.

**Results:**

Eight tasks achieved over 95% success rate. Task 5 (interrupted assessment of an existing resident) represented an outlier at 42% success (95% CI: 23.1–63.7). Seventeen distinct error types were recorded across tasks. Completion times varied considerably; Task 3 (assessing an existing resident's pain via video) had the longest geometric mean (335 s, 95% CI: 269.6–417.2), while log-out (Task 9) had the shortest (11 s, 95% CI: 6.6–18.4). Resident archiving (Task 7) recorded the highest number of attempts (17). The mean overall PSSUQ-PT score was 1.9 (SD: 1.06; 99% CI: 1.20–2.60), outperforming the normative mean of 2.82, with no overlap in 99% CIs. Seven usability problems were identified and linked with likely underlying usability defects.

**Conclusion:**

PainChek®-PT demonstrated effectiveness, efficiency and satisfaction in this context of use, supporting its intended use for conducting pain assessments in non-verbal PLWD. The robustness of the overall PSSUQ score indicates high perceived usability. The errors and usability problems identified in the simulated environment may be addressed through training and minor design refinements.

## 1 Introduction

Dementia represents a significant global health challenge of the twenty-first century. According to the World Health Organization, approximately 55 million people currently live with dementia worldwide, a figure projected to reach 153 million by 2050 (1). Dementia ranks as the seventh leading cause of death globally and constitutes a leading cause of disability and dependency among older adults, imposing a substantial socioeconomic burden on health systems and families (2).

Pain is a common symptom experienced by people living with dementia. A systematic review of 25 studies conducted in nursing homes reported that 10% to 80% of people with dementia experience significant acute or chronic pain, with higher prevalence among those with moderate or severe dementia (3). Despite this burden, another systematic review found that, in long-term care, dementia was the most frequent diagnosis associated with lower analgesic prescribing (4). This undertreatment may partly reflect difficulties in communicating symptoms, with pain instead presenting as behavioral change. Consistent with this, a retrospective study using data from Dementia Support Australia identified pain in 65.6% of referrals for behavioral and psychological symptoms of dementia (BPSD); pain was associated with more frequent and more severe neuropsychiatric behaviors, including aggression and agitation (5). In turn, BPSD may contribute to inappropriate antipsychotic use, which is associated with an increased mortality risk, cerebrovascular events and accelerated cognitive decline (6).

Unrecognized pain among people with dementia reflects multiple factors, including insufficient professional training in pain assessment and treatment, the complexity and subjectivity of existing observational tools, limited availability of guidelines, and organizational barriers, such as limited time (7, 8). Although observational tools aim to detect pain through non-verbal indicators, including body movement, changes in activity patterns and facial cues, systematic reviews report only moderate evidence supporting their psychometric properties (9) and highlight limitations, such as subjectivity and inter-observer variability (10).

Novel digital technologies offer promising avenues for developing user-friendly, valid, reliable and more objective tools for pain assessment. The PainChek® system is a software comprising four components to assess and monitor pain in people who cannot verbalize it, such as those with advanced dementia (11). A key component is the PainChek® point-of-care app, a regulatory-cleared medical device designed for easy use, leveraging a smart-device (phone or tablet) and AI for pain assessment and monitoring. During a 3-second facial scan, images captured by the smart-device camera are automatically analyzed by AI models to identify micro-expressions indicative of pain, based on the Facial Action Coding System (11). These data are then combined with other non-facial pain indicators inputted by the app user, through a series of digital checklists for five additional domains (Voice, Movement, Behavior, Activity and Body). The app automatically computes a pain score and assigns an intensity level: no pain (0–6), mild pain (7–11), moderate pain (12–15), or severe pain (≥16). Each assessment typically takes around three minutes, making the tool practical and feasible for care practice (12).

The PainChek® system supports informed dementia care and aligns with the principles of person-centered practice by enabling care tailored to an individual's needs, preferences and circumstances, while also supporting shared understanding and decision-making among care providers (13).

Usability evaluations of pain management mobile applications often lack methodological rigor, with reported limitations including the use of non-validated measurement instruments, reliance on single method approaches and an emphasis on commercial functionality rather than scientifically robust assessment (14). This gap is particularly important given the growing international adoption of the PainChek® app. As uptake expands across jurisdictions and settings, rigorous and well-reported usability evaluation is needed to support scale-up and to inform interpretation of effectiveness and implementation studies. Therefore, this study aimed to evaluate the usability of the Portuguese version of the PainChek® application (PainChek®-PT app) by formal caregivers in residential care facilities in Portugal.

## 2 Materials and methods

### 2.1 Study design and framework

The usability evaluation of the PainChek®-PT app was guided by a previously published protocol (15) aligned with ISO standards, specifically ISO 9241-11:2018 and ISO 25062:2025 (16, 17).

The study was conducted in two residential care facilities in the municipalities of Cascais and Setúbal, Portugal. Sites were selected using convenience sampling from facilities involved in ongoing research on pain management in non-verbal people living with dementia.

The evaluation was conducted in a controlled environment, employing a multi-method approach that combined testing and inquiry. For testing, the chosen techniques were task performance, observation of participants' performance and think-aloud (18), based on their established use in usability evaluation (19, 20). Inquiry relied on a questionnaire that included the Portuguese version of the Post-Study System Usability Questionnaire (PSSUQ-PT) (18).

This usability evaluation is reported in accordance with the checklist proposed by Martins et al. (20) and ISO 25062:2025; the completed checklists are provided in the Supplementary Material S1. This study was approved by the Egas Moniz School of Health & Science Ethics Committee, under the number 1367.

### 2.2 Sampling and recruitment of participants

Purposive sampling was used to select formal caregivers according to the following eligibility criteria: (i) ability to speak and read Portuguese; (ii) age over 18 years; (iii) access to a smartphone; (iv) employment in a residential care facility for at least three months; and (v) completion of prior training on the PainChek®-PT app.

In the protocol, two different rationales were presented: the completion-rate precision criterion yielded an estimated sample size of 17, whereas the problem-discovery model (assuming a per-problem occurrence probability of 0.10) indicates that 18 participants are needed to identify about 85% of usability problems. In this manuscript, the usability sample reported (*n* = 18) is justified using the problem-discovery rationale only (21).

Eligible formal caregivers were identified by residential care facility managers, who had received a study information leaflet. Individuals who agreed to participate were then sent the information leaflet with details about the study and the informed consent form by email. All participants signed the consent form prior to data collection.

### 2.3 Outcome measures

Effectiveness and efficiency were evaluated quantitatively based on the participants' task performance, using the outcome measures in Table 1 (16). To strengthen the rigor of the usability evaluation, we included additional outcome measures that were not specified in the published protocol: number and frequency of errors, appropriate action to the error message generated by the app, and number of attempts to perform the task steps.

**Table 1:** ISO definition of effectiveness and efficiency measures.

	Outcome measure	ISO 9241-11:2018 definition
Effectiveness	Task success rate	The extent to which users of the application are able to achieve the desired outcome.
	Number and frequency of errors	Actions or lack of action by the user while using the interactive system that leads to a result different from that intended by the manufacturer or expected by the user.
	Appropriate action after error messages generated by the application	Appropriate response to error messages in accordance with the guidelines.
Efficiency	Time to complete the task	For each task, this is the time measured between the user touching the screen after the instructions and completing the task.
	Number of attempts to perform the task steps	Effort required to correctly complete all steps of the task.

Outcome measures also included usability problems and defects. ISO 25062:2025 defines a usability problem as a “situation during use, resulting in poor effectiveness, efficiency, or satisfaction” (17). An example is difficulty accessing essential buttons. A usability defect is defined as an “attribute of the interactive system that leads to a usability problem” (17). Insufficient and/or poor-quality information in the user interface is considered usability defects. Identification of usability defects was not included in the original study protocol (15); this element was added to facilitate potential root-cause attribution and provide guidance for remediation.

### 2.4 Data collection procedures

Data collection took place after the training sessions, which were delivered on site by IBF at each residential care facility. The training consisted of two-hour sessions held approximately one week apart. The training materials, originally developed by PainChek®, were adapted into Portuguese by IBF, MF and MPG. The online, self-paced training (30–45 min) originally planned in the protocol (15) proved unfeasible within the available timeline due to the resources required for course development in Portuguese. The first face-to-face session covered the following topics: (i) pain in people with dementia; (ii) behaviors associated with pain; and (iii) key features of the PainChek®-PT app. The second session was hands-on and included two pain assessment exercises, one using automated facial analysis via video and one using manual facial analysis on two tablets with the PainChek®-PT app. The hands-on session concluded with a brief review to consolidate participants' learning.

Within three weeks after completing the training, usability data were collected in individual, face-to-face sessions lasting 13 to 50 min. The sessions were conducted by MF, at the time a Master of Pharmacy student who was not part of the application development team. MF had prior training in the collection and analysis of usability data of another application (https://www.vaprevention.org), using the same procedures and frameworks as in the current study. Only MF conducted the usability sessions to ensure consistency in study procedures across data collection.

Data were collected through task performance, concurrent think-aloud and observation notes in a controlled environment. Think-aloud was selected to identify usability problems by participants’ cognitive processes during task performance and clarifying how information is processed and applied in problem solving. The concurrent format was chosen because real-time verbalization captures thoughts, reasoning, and perceptions more accurately while tasks are being performed. This technique is also appropriate for small samples in a controlled environment and was therefore aligned with the objectives of the present study (15, 22).

A think-aloud script was developed, comprising nine tasks covering core features and the expected sequence of use of the PainChek®-PT app in routine use (Table 2), anchored in a fictional scenario of a person with dementia unable to verbalize pain. For example, Task 5 (Interrupted assessment) required caregivers to start a facial assessment in manual mode (i.e., recording observed facial pain indicators directly in the app rather than via video) and then interrupt and save the assessment to complete later. Each task had specific outcome measures, as described in the Supplementary Material S2 (PT) and 3 (EN). MF observed the participant's interactions while they completed the predefined tasks and documented any usability problems. The usability evaluation took place in a private, quiet room to minimize distractions and ensure a comfortable environment.

**Table 2:** Tasks included in the formal caregivers’ script.

Tasks	Task description
Task 1	Log in
Task 2	Register a new resident in the application
Task 3	Assess the pain of an existing resident using PainChek®, via video
Task 4	Review a previous pain assessment of a resident
Task 5	Interrupted assessment of an existing resident: delay in saving an assessment The task is subdivided into: ▪ 5.1 Choose a facial assessment in *Manual* mode ▪ 5.2 Interrupt the assessment, saving it to finish later
Task 6	Complete a previously unfinished assessment
Task 7	Archive a resident in the application
Task 8	Restore an archived resident in the application
Task 9	Log out

Participants performed the defined tasks on a Samsung Galaxy Tablet A9 running Android 14 in most sessions; when this was not available, an iPhone 11 with the iOS 17 operating system was used. After verbalizing their thoughts aloud in real time while performing each task, participants were invited to comment on their user experience. With participants' consent, the device screen and audio (i.e., participants' verbalization during the think-aloud protocol) were recorded using the devices’ built-in recording software to enhance the accuracy of subsequent data analysis.

This think-aloud approach enabled the collection of textual data, including usability problems, through direct observation of task performance.

After performing the tasks, participants were invited to complete a questionnaire on Google Forms to assess their experience, perceived usability and satisfaction. The questionnaire encompassed three sections: (i) sociodemographic characteristics; (ii) PSSUQ-PT and (iii) open-ended questions capturing perceived strengths and weaknesses of the application, and suggestions for improvement (Supplementary Material S4). The PSSUQ-PT (23) consists of closed-ended items rated on a 7-point Likert scale, from *strongly agree* (1) to *strongly disagree* (7), with lower scores indicating better usability. Evidence supports the PSSUQ-PT as a reliable and valid instrument, demonstrating satisfactory inter-rater reliability and construct validity (23). The 19 items cover system usefulness (items 1 to 8); information quality (items 9 to 15); interface quality (items 16 to 18) and overall satisfaction (item 19).

The think-aloud script was pre-tested with two healthcare professionals who were not included in the study sample. This pre-test assessed the clarity, consistency and comprehensibility of the instructions and tasks, and led to the removal of one task (“Set up a quick login PIN”), as the PIN persists after setup and the task can therefore be completed only once per device.

### 2.5 Data management and analysis

Effectiveness and efficiency measures were extracted by MF from the screen and voice recordings of the sessions. Table 3 presents the operationalization of these measures. The published protocol specified independent verification of extracted metrics. Owing to resource constraints, this was conducted for a random 10% of the sample as a quality control.

**Table 3:** Operationalization of effectiveness and efficiency measures.

Effectiveness measures	Operationalization
Task success rate	Number of participants who successfully completed the task (i.e., who performed all steps correctly and without any help) divided by the total number of participants Note: In task 5, only the number of participants who successfully completed both subtasks (5.1 and 5.2) is counted.
Error frequency	Total number of errors made by all participants during the task
Percentage of participants who made errors	Total number of participants who made an error divided by the total number of participants
Mean number of errors per participants	Total number of errors divided by the total number of participants
Appropriate action after error messages generated by the application	Number of participants who successfully completed the task and took appropriate action when an error message appeared
Inappropriate action after error messages generated by the application	Number of participants who successfully completed the task and took inappropriate action when an error message appeared

Efficiency measures	Operationalization
Time to complete the task	Mean total time of participants who successfully completed the task. Note: In task 5, only the average total time of participants who successfully completed both subtasks (5.1 and 5.2) is counted.
Attempts to perform the task steps	Number of times the participant had to repeat task steps until successful completion: ▪ 0 = completed correctly on the first attempt (no repetitions); ▪ ≥1 indicates that the participant needed more than one attempt, i.e., had to repeat one or more steps of the task until they were able to complete it. Note: In task 5, only the number of attempts by participants who successfully completed both subtasks (5.1 and 5.2) are counted.

Task success rates are reported as raw binomial completion percentages. To estimate population-level performance and account for the negative skew and the sample size (*n* = 19), 95% confidence intervals (CI) were calculated using the Adjusted-Wald method (24) via the MeasuringU calculator (https://measuringu.com/calculators/wald/).

Task times were analyzed assuming a log-normal distribution, as recommended by Sauro and Lewis (25). To mitigate the influence of outliers and the positive skew typically found in small usability samples, the data underwent a logarithmic transformation prior to calculating the 95% CI using the t-Student method via the MeasuringU calculator (https://measuringu.com/calculators/time_intervals/?sample=0#set). Results are reported using the geometric mean to represent the typical completion time, as it provides a more accurate estimate of central tendency for skewed time data than the arithmetic mean (25).

For PSSUQ-PT scores, missing responses were handled by replacing the item score with the mean of the participant's remaining (non-missing) item scores, as recommended (23). To enable a comparison with published normative values derived from IBM usability datasets (26), 99% CIs were computed using the MeasuringU confidence interval calculator for continuous data (https://measuringu.com/calculators/ci-calc/).

Relevant excerpts from the think-aloud sessions were transcribed. The transcripts, together with observation notes and the textual data from the questionnaire were screened by MF to extract usability problems; each was assigned a unique identifier and a clear description. Coding of usability problems was guided by the ISO 25062:2025 definition described previously (17). MF performed the initial coding and descriptions; these were subsequently reviewed with IBF and MPG to agree on the final wording and categorization. The resulting findings were then summarized in a usability problem matrix showing the distribution of problems across the sample. Two proportions were defined:

– Individual problem burden, calculated as the number of distinct usability problems encountered by a participant (A) divided by the total number of unique problems identified in the study, and converted into percentage.
– Participants affected, calculated as the number of participants who experienced a specific problem (B) divided by the total number of participants in the study (n), and converted into percentage.

After agreement was reached on usability problems, MF derived likely usability defects by assigning the most plausible system attribute contributing to the observed problems inferred from task performance, think-aloud and observation data. Descriptions were subsequently reviewed with IBF and MPG.

Suggestions emerging from the think-aloud sessions and questionnaires were thematically analyzed and listed.

Microsoft Excel was used to manage textual and numeric data and perform descriptive statistics. After analysis, all recordings were deleted and data were processed in anonymized form, in accordance with the terms of ethical approval of the study.

## 3 Results

### 3.1 Participant characteristics

Twenty formal caregivers participated in the usability evaluation of the PainChek®-PT app. One participant withdrew before completing all required tasks and was therefore excluded from the analysis. The final sample comprised 19 participants, exceeding the calculated target. Table 4 describes the participants' sociodemographic characteristics.

**Table 4:** Participants’ sociodemographic characteristics.

Sociodemographic characteristics	*n* = 19
Gender, *n* (%)	
Female	14 (73.7)
Male	5 (26.3)
Age in years, Mean (Standard Deviation)	39.4 (SD 11.2)
Level of education, *n* (%)	
Primary education	1 (5.3)
Secondary education	5 (26.3)
Bacheloŕs degree	10 (52.6)
Masteŕs degree	3 (15.8)
Professional category, *n* (%)	
Nurses	4 (21.1)
Psychologists	1 (5.3)
Physical therapists	4 (21.1)
Nurse assistants	7 (36.8)
Sociocultural animators	1 (5.3)
Occupational therapists	2 (10.5)
Professional experience in years, Mean (Standard Deviation)	8.0 (SD 7.5)

### 3.2 Effectiveness of PainChek®-PT app

Success rates were high across most tasks; eight out of nine tasks achieved over 95% success, while Task 5 (interrupted assessment of an existing resident) represented an outlier at 42%. Table 5 presents the observed success rates and the corresponding 95% CI (adjusted Wald).

**Table 5:** Success rate and 95% confidence intervals per task.

Tasks	Participants with task completed (n)	Observed success rate (%)	95% CI (%)
**Task 1**: Log in	19	100	[85.2–100]
**Task 2**: New resident	19	100	[85.2–100]
**Task 3**: Assess the pain	18	95	[73.5–100]
**Task 4**: Review a previous pain	18	95	[73.5–100]
**Task 5**: Interrupted assessment	8	42	[23.1–63.7]
5.1 Facial assessment in *manual mode*	16	84	[61.6–95.3]
5.2 Interrupt assessment	9	47	[27.3–68.3]
**Task 6**: Previously unfinished assessment	19	100	[85.2–100]
**Task 7**: Archive a resident	18	95	[73.5–100]
**Task 8**: Restore an archived resident	19	100	[85.2–100]
**Task 9**: Log out	18	95	[73.5–100]

A total of 17 error types were recorded. Task 3 recorded the highest error frequency, with four error types and a total of 27 occurrences. By contrast, Tasks 2 and 6 showed the highest accuracy, each involving a single error type and the fewest occurrences. Tasks 7, 8 and 9 also involved one error type each, but these errors were made by multiple participants. Details on per-task errors are presented in Table 6.

**Table 6:** Frequency and distribution of usability errors per task.

	Participants with error (n)	Participants with error (%)	Total error occurrences	Mean errors per user (Min; Max)
Task 1				
Filling in the username	3	15.8	3	0.16 (0; 1)
Filling in the password	4	21.1	4	0.21 (0; 1)
Total	7	Not applicable	7	0.37 (0; 2)
Task 2				
Filling in required fields	1	5.3	1	0.05 (0; 1)
Total	1	Not applicable	1	0.05 (0; 1)
Task 3				
Completing the pain assessment domains	7	36.8	7	0.37 (0; 1)
Intention to add features in *Video* mode	4	21.1	14	0.74 (0; 6)
Selecting the assessment timing (post-movement/rest)	3	15.8	3	0.16 (0; 1)
Selecting the “Numerical Rating Scale” option	2	10.5	3	0.16 (0; 2)
Total	16	Not applicable	27	1.42 (0; 7)
Task 4				
Choosing the desired pain assessment domain	4	21.1	4	0.21 (0; 1)
Selecting the resident	1	5.3	1	0.05 (0; 1)
Selecting the last assessment	1	5.3	1	0.05 (0; 1)
Total	6	Not applicable	6	0.32 (0; 2)
Task 5				
Evaluation not saved	7	36.8	7	0.37 (0; 1)
Appearance of characteristics in the “Face” domain, through *manual* mode	3	15.8	3	0.16 (0; 1)
Selection of the assessment timing	3	15.8	5	0.26 (0; 3)
Total	13	Not applicable	15	0.79 (0; 3)
Task 6				
Return to domains to be completed	1	5.3	1	0.05 (0; 1)
Total	1	Not applicable	1	0.05 (0; 1)
Task 7				
Choice of button used to archive the resident	9	47.4	11	0.58 (0; 2)
Total	9	Not applicable	11	0.5
Task 8				
Find the list of archive items	2	10.5	2	0.11 (0; 1)
Total	2	Not applicable	2	0.11 (0; 1)
Task 9				
Exit the application (wrong button)	4	21.1	4	0.21 (0; 1)
Total	4	Not applicable	4	0.21 (0; 1)

Appropriate action after the error message generated by the app was also evaluated (Table 7). Where applicable, most participants responded appropriately, particularly in Tasks 1, 2 and 3. Performance for Task 5 was less favorable: only four participants acted correctly, whereas seven did not.

**Table 7:** Participant responses to error messages per task.

	Task number								
Appropriate action after error message	1	2	3	4	5	6	7	8	9
Yes	6	1	8	NA	4	0	0	0	0
No	0	0	0	NA	7	0	0	0	0

NA, not applicable.

### 3.3 Efficiency of PainChek®-PT app

Task completion times varied significantly across the evaluation. The longest geometric mean completion time was observed for Task 3 (335 s), which involved assessing an existing resident's pain via video. Log-out (Task 9) had the shortest completion time, with a geometric mean of 11 s. Table 8 summarizes task completion times, including the geometric means and corresponding 95% CI (t-student) for all tasks.

**Table 8:** Mean completion times and 95% confidence intervals per task.

Task	Participants with task completed (n)	Geometric mean (seconds)	95% CI (seconds)
**Task 1**: Log in	19	63	[50.5–79.1]
**Task 2**: New resident	19	81	[65.8–99.8]
**Task 3**: Assess the pain	18	335	[269.6–417.2]
**Task 4**: Review a previous pain assessment	18	48	[38.1–60.1]
**Task 5**: Interrupted assessment	8	264	[177.1–392.7]
**Task 6**: Previously unfinished assessment	19	68	[53.9–85.3]
**Task 7**: Archive a resident	18	39	[25.8–58.1]
**Task 8**: Restore an archived resident	19	17	[10.3–26.8]
**Task 9**: Log out	18	11	[6.6–18.4]

Task 7 required the highest number of attempts to complete the task steps, whereas Task 6 required the lowest number, as shown in Table 9.

**Table 9:** Number of attempts to perform the task steps.

Tasks	Number of attempts (≥1)
**Task 1**: Log in	6
**Task 2**: New resident	3
**Task 3**: Assess the pain	10
**Task 4**: Review a previous pain	6
**Task 5**: Interrupted assessment	14
5.1 Facial assessment in manual mode	12
5.2 Interrupt assessment	2
**Task 6**: Previously unfinished assessment	1
**Task 7**: Archive a resident	17
**Task 8**: Restore an archived resident	8
**Task 9**: Log out	4

### 3.4 Usability problems and defects

A total of seven usability problems were identified; Table 10 details the relationship between observed problems and likely underlying usability defects. No likely usability defect was assigned to usability problem 6, in which participants were unable to set a preferred delay for resuming the assessment, because the fixed 30 min “Later” option reflects an intentional design characteristic: users are expected to return and complete the assessment within 30 min, after which the system prompts them to complete or delete the assessment as a reminder mechanism.

**Table 10:** Usability problems and defects identified .

Usability problem (user experience)	Likely usability defect
**Problem 1**: Users were unsure which field (username or password) was incorrect, leading to confusion and repeated failed login attempts.	Generic login error does not indicate whether username or password is incorrect (and provides limited guidance to resolve).
**Problem 2**: Users of the PT app encountered error messages in English that they could not readily understand, which delayed their next actions.	PT version shows some error messages in English.
**Problem 3**: Users struggled to find and view the information bubble within each assessment domain when they needed support.	Info/help bubble is hard to notice/access/read within domains (e.g., small, low-contrast, poorly signposted).
**Problem 4**: Users did not realize how to change domains and spent time trying different actions before succeeding.	Swipe gesture not signposted (no obvious affordance/instruction to swipe to change domains).
**Problem 5**: Users could not quickly find the “Later” option, delaying completion of the intended action.	“Later” button has low visibility/suboptimal placement, making it hard to locate quickly.
**Problem 6**: Users were unable to select their preferred “Later” duration and could only proceed with the default delay, which led to frustration or delayed completion	Not applicable.
**Problem 7**: Users struggled to find the “Log out” option and took longer than expected to sign out.	Log out is not easily discoverable due to its location/menu hierarchy.

Table 11 provides a task-level synthesis for Task 5, which had the lowest success rate. This synthesis links observed performance to the corresponding errors, usability problems and likely defects.

**Table 11:** Synthesis of task 5 performance, linking success rate, errors, usability problems and likely usability defects.

Task	Success rate	Specific errors	Related usability problems	Likely usability defect
Task 5: Interrupted assessment	42% [95% CI: 23.1%—63.7%]	(i) Evaluation not saved; (ii) appearance of characteristics in the “Face” domain through manual mode; (iii) selection of the assessment timing	Problem 5: Users could not quickly find the “Later” option, delaying completion of the intended action.	“Later” button had low visibility/suboptimal placement, making it hard to locate quickly.
			Problem 6: Users were unable to select their preferred “Later” duration and could only proceed with the default delay.	No likely usability defect was assigned because the fixed 30-minute delay was considered an intentional design characteristic.

Table 12 presents the distribution of problems across the sample and associated metrics. The most prevalent were problems 1 and 7, affecting 5 of the 19 participants (26%). Conversely, five problems were observed in only a single participant. Two participants (P6 and P11) experienced the highest individual problem burden (43%), encountering three of the seven problems, whereas eight participants completed their tasks without experiencing usability problems.

**Table 12:** Usability problem matrix.

	Usability problems								
	1	2	3	4	5	6	7	Total problems encountered (A)	Individual problem burden (A/7; %)
Participant (P) 1								0	0
P2	x							1	14
P3								0	0
P4	x							1	14
P5		x						1	14
P6	x		x				x	3	43
P7								0	0
P8								0	0
P9								0	0
P10								0	0
P11	x			x			x	3	43
P12								0	0
P13								0	0
P14							x	1	14
P15							x	1	14
P16	x							1	14
P17						x		1	14
P19							x	1	14
P20					x			1	14
**Total number of participants affected (B)**	5	1	1	1	1	1	5		
**Participants affected (B/19; %)**	26	5	5	5	5	5	26		

### 3.5 Suggestions from participants

Six suggestions were reported and are presented in Table 13. Five of the 19 participants made suggestions: one participant (P20) provided two suggestions, while the remaining four participants contributed one each.

**Table 13:** Suggestions reported by participants.

Participant code	Suggestion
P7	Send automatic notifications to remind the user to reassess pain.
P11	Strengthen the prominence of existing error alerts by e.g., increasing contrast, typographic size/emphasis, ensuring users notice the error.
P14	Provide additional training for users on how to use the application.
P17	By clicking on the “Later” button, the user could select how long the assessment would remain pending or, alternatively, leave it without a set deadline.
P20	Items belonging to each domain that may cause confusion should be better differentiated (e.g., “shouting” and “speaking loudly”) through clearer descriptions, practical examples, or adjustments to the nomenclature, enabling users to distinguish between them without repeatedly consulting the definitions.
	The “Later” button, associated with the feature of saving an incomplete assessment to resume later, is located at the end of all domains. Its location could be adjusted to be closer to the “Cancel” button.

### 3.6 Participant satisfaction

The mean overall PSSUQ score was 1.90 (SD: 1.06; 99% CI: 1.20–2.60). Table 14 presents the descriptive statistics and 99% CIs for the PSSUQ assessment of the PainChek®-PT app alongside the corresponding normative values.

**Table 14:** PSSUQ item and dimension scores for the PainChek-PT app compared to normative values (26).

	PainChek-PT usability assessment			Normative Values	
Item	Mean	SD	99% CI	Mean	99% CI
Q1	1.89	1.29	[1.04–2.74]	2.85	[2.60–3.09]
Q2	1.95	1.18	[1.17–2.73]	2.69	[2.45–2.93]
Q3	1.63	1.26	[0.80–2.46]	2.85	[2.58–3.11]
Q4	2.46	1.58	[1.42–3.50]	3.16	[2.86–3.45]
Q5	2.16	1.42	[1.22–3.10]	3.06	[2.79–3.34]
Q6	1.79	1.23	[0.98–2.60]	2.66	[2.40–2.91]
Q7	1.58	0.96	[0.95–2.21]	2.27	[2.07–2.48]
Q8	1.79	1.40	[0.87–2.72]	2.86	[2.54–3.17]
Q9	2.32	1.80	[1.13–3.51]	3.70	[3.36–4.05]
Q10	2.00	1.20	[1.21–2.79]	3.21	[2.93–3.49]
Q11	1.95	1.13	[1.20–2.70]	2.96	[2.65–3.27]
Q12	1.89	1.29	[1.04–2.74]	3.09	[2.79–3.38]
Q13	1.68	1.00	[1.02–2.34]	2.61	[2.37–2.86]
Q14	1.84	1.21	[1.04–2.64]	2.74	[2.46–3.01]
Q15	1.89	1.20	[1.10–2.68]	2.66	[2.41–2.92]
Q16	1.58	1.17	[0.81–2.35]	2.28	[2.06–2.49]
Q17	1.68	1.20	[0.89–2.47]	2.42	[2.18–2.66]
Q18	2.00	1.05	[1.31–2.69]	2.79	[2.51–3.07]
Q19	1.95	1.39	[1.03–2.87]	2.82	[2.55–3.09]
SysUse	1.91	1.10	[1.18–2.64]	2.80	[2.57–3.02]
InfoQual	1.94	1.08	[1.23–2.65]	3.02	[2.79–3.24]
IntQual	1.75	1.08	[1.04–2.46]	2.49	[2.28–2.71]
Overall	1.90	1.06	[1.20–2.60]	2.82	[2.62–3.02]

Mean item scores ranged from a minimum of 1.58 to a maximum of 2.46. Q7 (“Easy to learn”) and Q16 (“Liked using the interface”) shared the lowest mean of 1.58. Conversely, Q4 (“Complete tasks quickly”) yielded the highest mean of 2.46. Higher variability was observed in Q9 (“Clear error messages”), which had an SD of 1.80.

When asked what they most liked in the PainChek®-PT app, the most frequently cited aspect was ease of use (*n* = 10; 52.6%). Other favorable perceptions included the application's design (*n* = 1; 5.3%), its capacity to detect pain in people unable to communicate verbally (*n* = 3; 15.8%), its potential usefulness for individuals with limited experience in pain assessment (*n* = 1; 5.3%), the use of video to support assessment (*n* = 1; 5.3%) and the perception that the app represents a worthwhile investment in care and pain assessment for people living with dementia (*n* = 1; 5.3%).

Additionally, a small number of participants reported what they least liked about the use of the application: difficulty distinguishing specific behaviors, such as screaming and shouting (*n* = 2; 10.5%), the need to enter facial features manually (*n* = 1; 5.3%), and the perception that it was insufficiently fast and of limited utility for physicians and/or nurses, with a preference for traditional methods (*n* = 1; 5.3%).

## 4 Discussion

### 4.1 Interpretation of results, limitations and implications for practice and research

The overall objective of this study was to evaluate the usability of the Portuguese version of the PainChek®-PT app by formal caregivers in residential care facilities, addressing a gap in published evidence.

The systematic review by Almeida et al. (27) examined usability evaluation of pain-related mobile applications, highlighting variability in the usability elements evaluated, methods used and methodological rigor. Many studies relied on measurement instruments without demonstrated psychometric adequacy, with a large proportion failing to use tools with established validity and reliability. In addition, in this review usability evaluations prioritized user satisfaction and neglected the effectiveness and efficiency domains; only eight of 31 studies assessed all three domains. In contrast, the present study employed a standards-aligned approach, assessing effectiveness, efficiency, and satisfaction in accordance with ISO 9241-11:2018 and ISO 25062:2025 (16, 17). A multi-method approach was used, combining testing (task performance, observation, and think-aloud) with inquiry (18), based on a questionnaire with sound psychometric properties (23). Collectively, these choices strengthen rigor and credibility, as they allow integration of different data sources.

The effectiveness results were generally positive, with an average task success rate exceeding 90%. Specifically, the success rate for the interrupted assessment of an existing resident (Task 5: 42%, 95% CI: 23.1–63.7) was the lowest observed. Its CI remained below the lower bounds of CIs for all other tasks, where the lowest bound was 73.5%, indicating that even in a best-case scenario (63.7%), Task 5 performs worse than the worst-case estimates for other tasks. This statistical finding, combined with verbalized difficulties while performing Task 5, suggests that this workflow represents a friction point, which could be at least partly addressed with more scenario-based training.

Across all tasks, 17 types of errors were recorded. Their low number and dispersion suggest isolated difficulties rather than structural design flaws affecting multiple tasks and users. Task 3, corresponding to conducting a pain assessment with PainChek®-PT app, had a high success rate (95%, 95% CI: 73.5–100) but the highest error count (4 errors) and the highest frequency of error occurrence. Unlike predictably straightforward tasks, such as logging in and logging out, Task 3 is a multi-step process involving video assessment and manual inputs. Given the higher interaction points, the total number of errors likely reflects the workflow and limited user familiarity with the process.

Efficiency was assessed by the average time to complete each task and the number of attempts required for individual task steps. The average completion time for assessing the pain of a resident using the PainChek®-PT app via video (Task 3) was 5.6 min, with a 95% CI indicating that the estimate ranges from 4.5 to 7.0 min, which is longer than the 3 min reported for routine PainChek® assessment. This discrepancy is likely attributable to the simulation context and, as mentioned above, participants' limited familiarity with the process. Another contributing factor may be that participants had limited experience with observational pain assessment tools, predominantly using numerical rating scales. Moreover, completion times across tasks were likely inflated by the concurrent think-aloud protocol, which required participants to verbalize their thoughts while performing the tasks. Furthermore, Task 3 was the most error-prone task, and the observed errors and recovery attempts plausibly increased completion time. These factors may also explain why relatively simple tasks, such as logging out (Task 9, 11 s, CI: 6.6–18.4), required more time than would normally be expected in routine use. Overall, completion time for each task appears acceptable, although interpretation is limited by the absence of published usability data of the PainChek® app for comparable tasks.

Regarding attempts to complete task steps, Task 7, saving a resident in the app, had the highest number of attempts. Limited experience with the app can explain this pattern: 8 of the 19 participants selected the wrong save option, indicating difficulty identifying the correct step. By comparison, Task 6, completing an unfinished assessment, required the fewest attempts, likely because it repeated steps already practiced elsewhere.

The qualitative findings were consistent with the task-performance data. Seven usability problems and six improvement suggestions were identified. The most frequent were problems 1 and 7. Problem 1 reflected users' uncertainty about which login field (username or password) was incorrect, leading to confusion and repeated failed login attempts. Although labelled as a problem, participants in Task 1 typically took appropriate corrective actions. Usability defects should be interpreted as potential underlying design contributors inferred from observed use in this study, rather than defects established through formal inspection. Nevertheless, they can inform iterative refinement; for example, the occasional incomplete translation observed in the evaluated version has reportedly been addressed in a subsequent app update. The merit of this approach lies in providing the PainChek® development team with data that can be interpreted transparently within the global usability datasets.

Most suggestions focused on enhancements to the application. One participant highlighted the need for additional training, reinforcing the importance of ongoing support noted above. This need is further supported by the errors observed across tasks and may also help explain the likely usability problems, which point to specific aspects that should be reflected in the refinement of training (e.g., prioritizing error-prone steps). The observed usability problems may also reflect limitations in the training provided, particularly a potentially insufficient practical component. For example, in problem 4, several participants did not immediately recognize how to change domains. Although this aspect was addressed during training, it is possible that participants did not have sufficient opportunities to practice it. Increasing hands-on practice during training may therefore improve users' proficiency. Additionally, usability evaluation was conducted up to three weeks after the training, during which participants did not have access to the app. The time between training and assessment varied due to participant's availability, ranging from one day to three weeks, which may have influenced performance.

The PSSUQ-PT yielded an overall mean of 1.9 (SD: 1.06; 99% CI: 1.20–2.60), on a scale from 1 (most positive) to 7 (least positive); this overall score is lower than the normative mean (2.82). The PainChek®-PT app scores were lower than normative values across all dimensions**—**System Usefulness (1.91), Information Quality (1.94), and Interface Quality (1.75)**—**as well as in the overall satisfaction item, Q19 (1.95). In the Information Quality dimension and the Overall score, the 99% CI for the app did not overlap with the normative 99% confidence intervals. The lack of overlap at such a conservative confidence level indicates that the app's superior usability ratings are statistically robust and unlikely to be the result of sampling error. Interface Quality was the best-performing dimension, which is consistent with the normative pattern. This finding suggests that, despite occasional difficulty locating certain buttons, the interface was generally perceived as pleasant and that its features aligned with users' expectations. It should be noted that comparison with normative values places the favorable PSSUQ scores in perspective but should not be interpreted as definitive superiority of the PainChek®-PT app. These normative values derive from pooled usability studies involving mostly non-health software systems with different contexts of use (26), therefore, they serve as a contextual reference rather than a strict benchmark.

In this study, usability was evaluated after training because prior training is a prerequisite for use PainChek®-PT in practice. Providing training prior to usability evaluation is endorsed by ISO 9241-11:2018 (16) and has been followed by others (28, 29), particularly when digital products require technical and procedural knowledge. This approach may, however, raise the criticism of whether results reflect usability or learnability. While observed difficulties may reflect the short duration and density of training content, the findings should be interpreted as reflecting usability for two reasons: purpose and evaluation procedures. First, the purpose of usability evaluation is to examine whether specified users could complete specified tasks effectively, efficiently, and satisfactorily within a defined context of use (16). Second, in the present study, the evaluation procedures were conducted separately from the training sessions and progressed from didactic demonstration and feedback to independent task performance in a simulated environment at different time points. Future usability evaluations should nevertheless benefit from incorporating a more structured learning assessment to ensure that participants have acquired a minimum required technical knowledge before proceeding to usability evaluation.

Several limitations should be considered when interpreting the overall usability findings, particularly because usability outcomes are context-dependent, reflecting the interaction between users, tasks and the environment (30). The study was conducted in only two private residential care facilities, which may limit transferability to Portuguese facilities with different staffing, resources, and service provision. In addition, nurse assistants were underrepresented relative to their weight in the residential care workforce. This affects the representativeness of the sample if nurse assistants are intended to be primary users of the PainChek-PT® app in practice, potentially leading to an overestimation of usability outcomes.

Although sample size was consistent with a commonly used problem-discovery rationale for usability studies, no formal saturation analysis was undertaken. The final analytical sample slightly exceeded the estimated target for identifying approximately 85% of usability problems, but this does not imply exhaustive problem discovery.

Usability was assessed in a controlled simulation environment with a predefined task sequence that mimicked actions performed in practice during pain assessment. Although this approach improves standardization, it did not reproduce the conditions of routine practice; contextual factors may affect the effectiveness and efficiency of the application under real-world conditions. Therefore, future work should complement these findings with real-world usability evaluations. In addition, ongoing improvements should be supported through mechanisms that capture real-world user feedback and issues, such as structured user reporting and periodic user surveys.

Another limitation of this study is that the identification and categorization of usability problems and likely defects were initially conducted by a single team member and subsequently discussed with two other members; this precluded inter-rater agreement estimates and lends itself to individual biases and omissions. Moreover, independent verification of extracted metrics was conducted for only 10% of the sample. Although no discrepancies were identified in this subsample, this procedure provides only partial reassurance regarding measurement accuracy and does not exclude undetected error in the remaining data. Nevertheless, the overall pattern of findings showed internal coherence across task success, errors, attempts, task completion times, and the usability problems identified during observation. For example, Task 5 had the lowest success rate and poorer performance after error messages; Task 3 had the highest error count and longest completion time; and Task 7 had the highest number of attempts. This overall coherence supports data accuracy.

An additional direction for future research is to assess participants' digital competence in mobile health usability evaluations, as user characteristics can influence interaction performance. Tools such as the Digital Competence Framework for Citizens (DigComp) map key competences across five areas: information and data literacy; communication and collaboration; digital content creation; safety and problem solving (31). Where sample size permits, future studies could explore associations between digital competence and measures of effectiveness and efficiency (31). Notably, there is no established evidence for the use of this tool specifically in usability studies.

### 4.2 Implications for person-centered practice

The Person-Centered Practice Framework (PCPF) is widely cited and is grounded in its capacity to translate the normative aspirations of person-centeredness into an actionable and evaluable approach to care delivery. The framework operationalizes person-centeredness by providing a shared language and a structured, middle-range theoretical model with empirically observable components. It also extends beyond individual encounters to emphasize the development of healthful, flourishing workplace cultures in which staff are supported, valued and empowered. By explicitly addressing prerequisites, the care environment, person-centered processes and outcomes, the framework is well suited to the complexity of contemporary healthcare and offers a systematic approach for how practice change is expected to occur (13).

Usability findings yield important implications for several components of the PCPF. First, findings indicate that, despite prior training, difficulties persisted in specific tasks, and some participants requested additional support. This suggests that the implementation of PainChek® may require ongoing investment in capability development to ensure autonomous and consistent use, thereby strengthening the prerequisites domain. Understanding how these capabilities and related prerequisites vary across caregivers is therefore essential to tailor training and implementation strategies that support person-centered practice.

Second, the findings have implications for the care environment, which within the PCPF encompasses organizational systems of support, effective relationships and conditions that enable innovation. Digital tools must be aligned with real-world workflows and contextual constraints; otherwise, cognitive load, time demands and interruptions may increase, particularly in settings characterized by heterogeneous caregivers' profiles. Identifying usability problems provides an opportunity to optimize the Portuguese version of the PainChek®-PT app and reduce friction, thereby better supporting this component of the PCPF and its enabling conditions.

Usability was assessed in a controlled environment using simulation, which has implications for understanding person-centered processes within the PCPF. Simulation does not fully capture how caregivers actually enact authentic engagement and sympathetic presence when using the PainChek®-PT app during everyday interactions with residents. While the findings suggest that the application is usable under standardized conditions, further research is needed to explore how its use is integrated into real care encounters in Portugal, and whether it supports or constrains the quality of relational practice that is central to person-centered care.

Establishing that the PainChek®-PT app is effective, efficient and satisfying to use is a necessary foundation for achieving outcomes such as better recognition and management of pain, reduced distress and agitation, and enhanced quality of life for residents living with dementia. Future research should therefore move beyond usability metrics to examine how the implementation of the PainChek®-PT app influences pain-related outcomes and indicators of a person-centered care culture.

## Data Availability

The datasets presented in this article are not readily available because The datasets generated and/or analyzed during the current study cannot be made openly available because participants did not provide written consent for open sharing. Requests to access the datasets should be directed to Requests to access the datasets should be directed to Egas Moniz Ethics Committee at comissaoetica@egasmoniz.edu.pt.
