# Supplementary material for: Towards person-centered pain management in dementia: usability of a digital medical device in Portuguese residential care facilities
Source: Front Health Serv. 2026 Jun 24;6:1829082. doi: 10.3389/frhs.2026.1829082 (PMC13342171; doi:10.3389/frhs.2026.1829082)
Supplement: Supplementary file 1 [file Datasheet1.pdf]

Agreement on the items for usability evaluation involving users (Martins et al. (2023))

**Usability assessment moderator**	
Determine the number of usability assessment moderators.	Page: 5<br>Line: 178
Provide the rationale used to establish the number of usability assessment moderators.	Page: 5<br>Line: 181
Specify as inclusion criteria having previous experience with usability evaluation with users or consider adequate training and provide details of the training plan.	Page: 5<br>Line: 178-181
Detail inclusion and exclusion criteria other than previous experience (e.g., academic background or age).	Page: 5<br>Line: 178-181
Specify whether the usability assessment moderators are external to the service or product development team.	Page: 5<br>Line: 178-181
Specify if observers are included, define their responsibilities, and collect their characteristics (e.g., gender, academic background, and previous experience in usability evaluation).	NA
Detail the usability assessment moderators' characteristics that should be collected (e.g., gender, academic background, and previous experience conducting usability evaluation).	Page: 5<br>Line: 178-181
**Participants**	
Determine sample size (i.e., the total number of participants involved in the evaluation).	Page: 8<br>Line: 420
Provide a rationale to establish the sample size.	Page: 4<br>Line: 122-126
Provide clear inclusion and exclusion criteria (e.g., profile definition including age, gender, educational level, digital literacy, and previous experience using the product or service being evaluated).	Page: 4<br>Line: 118-121
Provide sampling methods (e.g., random, systematic, cluster, convenience, and snowball).	Page: 4<br>Line: 118
Indicate the setting of participants' recruitment (e.g., community and hospital).	Page: 4<br>Line: 104-106
Detail clinical conditions (if relevant for the study) (e.g., asymptomatic or with a specific clinical condition or from a specific group—occupational group, the severity of the clinical condition, disabilities, cognitive impairment).	NA
Detail the participant's characteristics that should be collected (such as age, gender, educational level, and digital literacy).	Supplementary Material 4
**Usability evaluation method and usability evaluation technique**	
Specify whether a combination of usability evaluation methods was used (e.g., using both inquiry and test methods).	Page: 4<br>Line: 107-111
Specify whether a combination of usability evaluation techniques was used (e.g., for the inquiry method, combine the questionnaire and interview techniques).	Page: 3<br>Line: 108-111

Provide the rationale for the choice of usability evaluation methods and techniques.	Page: 5/6<br>Line: 108-111/<br>194-195
Describe the usability evaluation methods and techniques used and how they are implemented.	Page: 5/6<br>Line: 164-170/<br>167-290
When using measuring instruments such as scales or questionnaires, give indicators of their validity and reliability.	Page: 6<br>Line: 167-290
Describe the data analysis plan for both quantitative and qualitative data.	Page: 6/7<br>Line: 293-349
**Tasks**	
Provide a detailed description of tasks or present the session script.	Supplementary Material 2/3
Indicate the total number of tasks.	Page: 6<br>Line: 259
Detail the task-related outcomes and how they are measured (e.g., task completion and duration and number of errors).	Supplementary Material 2/3<br>Page: 7/8<br>Line: 225/293-349
Detail the conditions for carrying out the tasks (e.g., with or without supervision, individually or in the group, with or without a period for familiarization with the digital product or service).	Page: 5/6<br>Line: 167-276
Detail the instructions to participants and the way they are presented (e.g., verbally, written, and both) and registered (e.g., audio, video, screen recorder, and notes from an observer).	Page: 5/6<br>Line: 167-276
**Usability evaluation environment**	
Justify the choice of the usability evaluation environment (e.g., lab or field test and remote or face-to-face test).	Page: 4<br>Line: 94-96
Specify usability evaluation environment requirements (e.g., recording equipment or observer room availability).	Page: 6<br>Line: 269-274;
Detail the procedures to make the usability evaluation environment safe and comfortable for the participants.	Page: 6<br>Line: 266-267

Checklist of content elements for a usability evaluation report (ISO 25062:2025)

**Executive summary**	
Name and description of the object evaluation	Page: 1<br>Line: 21-26
Summary of method(s) and the procedure	Page: 1<br>Line: 28-55
Summary of results including key findings	Page: 2<br>Line: 57-64
Recommendations and related conclusions (Optional)	Page: 2<br>Line 66-70
**Description of the object of evaluation**	
Formal name and release or version of the object	Page: 3<br>Line: 27
Brief description of the object	Page: 3<br>Line: 27-38
Purpose of the object	Page: 3<br>Line: 27-38
Intended use of the object	Page: 3<br>Line: 27-38
Parts of the object that were evaluated (Optional)	NA
Prior usability evaluation reports summaries (Optional)	NA
**Purpose of the usability evaluation**	
Reasons for which the evaluation was conducted	Page: 3<br>Line: 90-96
Reasons that only specific parts of the object were (Optional)	NA
**Evaluation methodology**	
Type(s) of evaluation used	Page: 4<br>Line: 107-111
**Evaluators**	
Total number of evaluators	Page: 5<br>Line: 177
Qualification of each evaluator and their affiliation (if external)	NA
Role of each evaluator during the evaluation	Page: 5<br>Line: 178-182
**Evaluation participants**	
Total number of evaluation participants	Page: 8<br>Line: 420
Planned key characteristics of evaluation participants	Page: 4<br>Line: 118-121
Actual key characteristics of the evaluation participants in the evaluation	Page: 9<br>Table 4
**Tasks used for evaluation**	
Tasks used for evaluation	Page: 6<br>Line: 259-265;<br>Table 2

Tasks scenarios	Supplementary Material 2 and 3
Criteria for successful task completion or task abandonment for each task	Page: 7<br>Table 3
Rational for the tasks used	Page: 6<br>Line: 259-261
**Evaluation environment**	
Description of the technical environment including hardware and/or used	Page: 6<br>Line: 269-274
Physical and social, cultural and organizational environment in which the evaluation took place	Page: 3/6<br>Line: 104-106/266-267
Description of the resources used including relevant hardware, software, and materials	Page: 6<br>Line: 269-274
Description of the administration tools used to control the evaluation or to record data (Optional)	NA
Data to be collected during the evaluation (usability defects, usability findings, performance data, qualitative data, or quantitative data)	Page: 5/6<br>Line: 152-164/ 183-258
Conformity assessment scheme (Optional)	NA
**Data analysis and results**	
**Data analysis**	
Approach used for the analysis of collected data	Page: 7<br>Line: 314-349
Differences in planned and collected data (Optional)	NA
Portion of data not used in the analysis (Optional)	NA
Data scoring	Page: 5<br>Line: 152-164<br>Page:6<br>Line: 281-286<br>Page 7: 336-340
Statistical analyses used to analyse the data	Page: 7<br>Line: 314-349
**Reported results**	
**Usability findings**	
Identified usability defects	Page: 9<br>(Table 10)
Potential usability problems	NA
Observed usability problems	Page: 9<br>(Table 10)
Recorded performance data	Page: 8<br>Line: 427-460
User-reported qualitative perceptions and responses (Optional)	Page: 9<br>Line: 554-556
User-reported quantitative ratings of perceptions and responses	Page: 9<br>Line: 560-737

**Recommendations** (Optional)	NA
**Conclusions** (Optional)	Page: 20<br>Line: 488-492
**Appendix**	
Evaluation protocol (Optional)	Page: 4<br>Line: 102
Sequence of organizational activities for conducting the evaluation (Optional)	NA
Independent variables (Optional)	NA
Predefined evaluation criteria (Optional)	NA
General instructions given to the participants (Optional)	Supplementary Material 2 and 3
Specific instructions on tasks (Optional)	Supplementary Material 2 and 3
**Additional content on ethics and intellectual property**	
Consent form (Optional)	Page 4<br>Line: 114-115; 127-149
Data management policy (Optional)	NA
Compensation policy (Optional)	NA
Use of artificial intelligence as part of generating the evaluation report (Optional)	NA
