# Supplementary material for: Towards person-centered pain management in dementia: usability of a digital medical device in Portuguese residential care facilities
Source: Front Health Serv. 2026 Jun 24;6:1829082. doi: 10.3389/frhs.2026.1829082 (PMC13342171; doi:10.3389/frhs.2026.1829082)
Supplement: Supplementary file 2 [file Datasheet2.pdf]

Guião Think-aloud

<u>Amostra</u>: Cuidadores Formais

Tarefa 1 – <u>Entrar na aplicação</u>:

**Pré-condições**	• Smartphone com a aplicação PainChek instalada;<br>• Criar um login de acesso (utilizador fictício);<br>o Nome do utilizador: margarida.XXXXXX@xxxx<br>o Palavra-passe: ERPI#1teste<br>• Ter impressa a informação de login;<br>• Ter acesso à Internet.
**Prompts**	Hoje assume o papel da Maria Ferreira que é profissional na Estrutura Residencial para Pessoas Idosas. Ainda não tem um login de acesso na aplicação PainChek. Pode criar um login de acesso à aplicação?<br><br>***Fornecer dados de login de acesso à aplicação***
**Atividade avaliada**	Iniciar sessão na aplicação móvel PainChek, utilizando uma determinada conta de utilizador:<br><br>1. Introduzir o nome de utilizador fictício<br>2. Selecionar *Seguinte*<br>3. Introduzir a palavra-passe<br>4. Selecionar *Iniciar Sessão*
**Medidas de resultado**	(1) Taxa de sucesso de execução da tarefa; (2) tempo para completar a tarefa; (3) número de erros e frequência de erros; (4) ação apropriada após as mensagens de erro geradas pela aplicação; (5) número de tentativas de execução dos passos na tarefa.

Tarefa 2 – Registar um novo residente na aplicação:

**Pré-condições**	• Aplicação deve ter alguns residentes já registados: residentes na generalidade; "meus residentes"; alguns destes residentes têm avaliações e outros não;<br>• Ter impressa a informação sobre a residente: Maria José Santos, nascida a 5 de julho de 1951, feminino, residente em Lado do Lago, Ala de Demências, Quarto 3. (**ALTERAR O NOME A CADA TESTE**)
**Prompts**	Esta é a Maria José Santos **(NOME ALTERADO A CADA TESTE)** é uma nova residente. A Maria ainda não está registada na aplicação PainChek. Pode registá-la na aplicação e avisar-me quando tiver terminado? Aqui estão as informações da residente.<br>***Apresentar ao participante um documento impresso com as informações do residente.***
**Atividade avaliada**	É pedido aos utilizadores que adicionem um residente com todos os passos incluídos neste processo:<br>1. Iniciar a sessão na aplicação (uma das seguintes opções):<br>→ Opção 1: Selecionar *Adicionar novo residente*<br>→ Opção 2: Selecionar *Os meus residentes* e, em seguida, selecionar *Adicionar novo*<br>→ Opção 3: Selecionar *Residentes* e, em seguida, selecionar *Adicionar novo*.<br>2. Preencher todos os campos do formulário (Nome, Apelido, Sexo, Data de nascimento, Localização e nome da residência e Quarto).<br>3. Selecionar *Guardar*.<br>4. O novo perfil deve ser encontrado no painel de controlo, na secção "Residentes".
**Medidas de resultado**	(1) Taxa de sucesso; (2) tempo para completar a tarefa; (3) número de erros e frequência de erros; (4) ação apropriada após as mensagens de erro geradas pela aplicação; (5) número de tentativas de execução dos passos na tarefa.

Tarefa 3 – Proceder à avaliação da dor com a PainChek, através de vídeo: realizar a avaliação do residente existente e realizar uma avaliação facial através de vídeo:

**Pré-condições**	A entrevistadora assume o papel de ator é instruído para:<br>• Agarrar no ombro;<br>• Apertar os olhos e mostrar os dentes;<br>• Soluçar e suspirar;<br>• Responder de forma brusca às questões colocadas, como por exemplo, "deixa-me em paz" ou "não sei".
**Prompts**	A Maria José Santos **(NOME ALTERADO A CADA TESTE)** tem tido queixas de dores no ombro. Enquanto profissional, gostaria de avaliar o nível de dor com a aplicação PainChek, utilizando o modo de *Vídeo*. Pode proceder a esta avaliação e avisar-me quando tiver terminado e guardado a avaliação?
**Atividade avaliada**	1. Abrir o perfil de residente da Maria José Santos (optar por uma das seguintes opções):<br>→ Opção 1: Selecionar *Residentes*, procurar Maria José Santos na lista, selecionar *Maria José Santos*<br>→ Opção 2: Selecionar *Os meus residentes*, procurar Maria José Santos na lista, selecionar *Maria José Santos* (apenas disponível se o residente for atribuído ao utilizador pelo participante na tarefa 3)<br><br>2. Selecionar *Avaliar a dor*<br>3. Selecionar uma das seguintes opções:<br>→ Opção 1: Ativar *Em repouso*<br>→ A opção 2: Ativar *Pós-movimento* não deve ser escolhida porque a Maria está sentada<br><br>4. Selecionar *Próximo*<br>5. Ativar *Vídeo*<br>6. Apontar a câmara do smartphone para o rosto do residente<br>7. Aguardar que o ecrã fique ativo

	8. Selecionar *Iniciar análise*<br>9. Quando terminar, deslizar para a esquerda<br>10. Preencher as restantes 5 listas de verificação de domínios<br>11. Selecionar *Exibir resumo*<br>12. Selecionar *Guardar*<br>13. Selecionar *Guardar* na janela pop-up
**Medidas de resultado**	(1) Taxa de sucesso; (2) tempo para completar a tarefa; (3) número de erros e frequência de erros; (4) ação apropriada após as mensagens de erro geradas pela aplicação; (5) número de tentativas de execução dos passos na tarefa.

Tarefa 4 – Rever uma avaliação anterior da dor de um residente na aplicação:

**Pré-condições**	• Carlos Prata está disponível na aplicação;<br>• O perfil do Carlos Prata deve conter uma avaliação prévia com os seguintes dados:<br>o Domínio Comportamento, registo de "Agressivo" e "Confuso"<br>• Carlos Prata deve estar atribuída à lista de residentes do utilizador.
**Prompts**	Um dos seus colegas está a perguntar pelo seu residente Carlos Prata, pois está preocupado com o seu comportamento recente.<br><br>Pode, por favor, abrir a última avaliação do residente e descrever o comportamento do Carlos Prata para atualizar o seu colega?
**Atividade avaliada**	1. Abrir o perfil do residente Carlos Prata (optar por uma das seguintes opções):<br>→ Opção 1: Selecionar *Residentes*, procurar Carlos Prata na lista, Selecionar Carlos Prata<br>→ Opção 2: Selecionar *Os meus residentes*, procurar Carlos Prata na lista, Selecionar Carlos Prata<br><br>2. Selecionar a última avaliação<br>3. Selecionar a lista de verificação do domínio *Comportamento*

	4. Transmitir ao entrevistador que o residente estava agressivo e confuso
**Medidas de resultado**	(1) Taxa de sucesso; (2) tempo para completar a tarefa; (3) número de erros e frequência de erros; (4) número de tentativas de execução dos passos na tarefa.

Tarefa 5 – Avaliação interrompida dos residentes existentes: atraso em guardar uma avaliação:

**Pré-condições**	• António Dias está disponível na aplicação;<br>• António Dias está atribuído à lista de residentes do utilizador;<br>• A entrevistadora assume o papel de ator é instruído para:<br>→ Manter a mão sobre a cara, tapando um olho, para impedir o reconhecimento facial;<br>→ Vocaliza que está a sentir dores: "Ai, a minha cabeça!" ou algo semelhante.
**Prompts**	Este é o residente António Dias, que caiu recentemente e sofreu um traumatismo craniano ligeiro e pretende fazer uma avaliação da dor. Pode proceder a esta avaliação e avisar-me quando tiver terminado?<br>*Interromper o participante quando o participante estiver a completar o domínio do Movimento, e continuar o Prompt*<br>Necessita de responder a uma emergência ao fundo do corredor, mas quer continuar a avaliação da dor mais tarde. Gostaria de adiar a avaliação para poder voltar a mesma mais tarde. Pode fazer isso e avisar-me quando tiver terminado?
**Atividade avaliada**	1. Abrir o perfil do residente António Dias (optar por uma das seguintes opções):<br>→ Opção 1: Selecionar *Residentes*, deslocar-se para ou procurar António Dias na lista, Selecionar *António Dias*

	→ Opção 2: Selecionar *Os meus residentes*, deslocar-se para ou procurar António Dias na lista, Selecionar *António Dias*<br><br>2. Selecionar *Avaliar dor*<br>3. Selecionar uma das seguintes opções:<br>→ Opção 1: Ativar *Em repouso*<br>→ Opção 2: Ativar *Pós-movimento*<br><br>4. Selecionar uma das seguintes opções:<br>→ Opção 1: Ativar *Manual*<br>→ Opção 2: Ativar *Vídeo*, depois passar para *Manual* quando a análise de vídeo não funcionar<br><br>5. Continuar com as listas de verificação (domínios da Voz e do Movimento)<br><br>***INTERRUPÇÃO***<br><br>6. Deslizar para a última página (lista de verificação do domínio Corpo)<br>7. Selecionar *Mostrar resumo*<br>8. Selecionar *Mais tarde*<br>9. Selecionar *Mais tarde* na janela pop-up
**Medidas de resultado**	(1) Taxa de sucesso; (2) tempo para completar a tarefa; (3) número de erros e frequência de erros; (4) ação apropriada após as mensagens de erro geradas pela aplicação; (5) número de tentativas de execução dos passos na tarefa.

Tarefa 6 – Completar uma avaliação não concluída anteriormente:

**Pré-condições**	Com base nos cenários anteriores: concluir a avaliação incompleta do residente António Dias.
**Prompts**	Retomar a avaliação da dor do residente António Dias, com o objetivo de finalizar à avaliação.
**Atividade avaliada**	1. Abrir o perfil do residente António Dias (optar por uma das seguintes opções):<br>→ Opção 1: Selecionar *Residentes*, deslocar-se para ou procurar António Dias na lista, Selecionar *António Dias*<br>→ Opção 2: Selecionar *Os meus residentes*, deslocar-se para ou procurar António Dias na lista, Selecionar *António Dias*<br>2. Selecionar uma das seguintes opções:<br>→ Opção 1: Selecionar a avaliação de cor laranja (não concluída)<br>→ Opção 2: Selecionar *Avaliar dor* e, em seguida, selecionar *Atualizar*<br>3. Selecionar um dos domínios incompletos, para voltar à lista de verificação associada<br>4. Completar os restantes domínios (Comportamento, Atividade, Corpo)<br>5. Selecionar *Exibir resumo*<br>6. Selecionar *Guardar*<br>7. Selecionar *Guardar* na janela pop-up
**Medidas de resultado**	(1) Taxa de sucesso; (2) tempo para completar a tarefa; (3) número de erros e frequência de erros; (4) ação apropriada após as mensagens de erro geradas pela aplicação; (5) número de tentativas de execução dos passos na tarefa.

Tarefa 7 – <u>Arquivar um residente na aplicação</u>:

**Pré-condições**	• Ana Tomé está disponível como residente ativa na aplicação;<br>• Ana Tomé está atribuída à lista de residentes do utilizador.
**Prompts**	A residente Ana Tomé vai mudar-se para outro estabelecimento. Pode, por favor, retirar a residente da lista de residentes e avisar-me quando tiver terminado?
**Atividade avaliada**	1. Abrir o perfil da residente Ana Tomé (optar por uma das seguintes opções):<br>→ Opção 1: Selecionar *Residentes*, deslocar-se para ou procurar Ana Tomé na lista, Selecionar Ana Tomé<br>→ Opção 2: Selecionar *Os meus residentes*, deslocar-se para ou procurar Ana Tomé na lista, Selecionar *Ana Tomé*<br>2. Selecionar 3. Selecionar *Arquivo*
**Medidas de resultado**	(1) Taxa de sucesso; (2) tempo para completar a tarefa; (3) número de erros e frequência de erros; (4) ação apropriada após as mensagens de erro geradas pela aplicação: (5) número de tentativas de execução dos passos na tarefa.

Tarefa 8 – <u>Restaurar um residente arquivado na aplicação</u>:

**Pré-condições**	Com base no cenário anterior, pretende-se restaurar o arquivo da residente Ana Tomé.
**Prompts**	A residente Ana Tomé vai regressar à residência Lado do Lago. Pode repor o perfil da mesma na lista de residentes?
**Atividade avaliada**	1. Selecionar uma das seguintes opções:<br>→ Opção 1: Selecionar *Residentes*<br>→ Opção 2: Selecionar *Os meus residentes*

	2. Selecionar *Arquivo*<br>3. Selecionar ⋮ à direta do perfil de residente Ana Tomé na lista<br>4. Selecionar *Restaurar*<br>5. Selecionar *Sim*<br>6. *Selecionar <Home>* para voltar à página inicial
**Medidas de resultado**	(1) Taxa de sucesso; (2) tempo para completar a tarefa; (3) número de erros e frequência de erros; (4) ação apropriada após as mensagens de erro geradas pela aplicação; (5) número de tentativas de execução dos passos na tarefa.

Tarefa 9 – Terminar sessão na aplicação:

**Pré-condições**	Com base no cenário anterior, o utilizador pretende sair da aplicação.
**Prompts**	Quando estiver concluído: Pode terminar a sessão e fechar a aplicação?
**Atividade avaliada**	1. Abrir o Menu selecionando o ícone ≡<br>2. Selecionar *Terminar Sessão* ou *Sair*
**Medidas de resultado**	(1) Taxa de sucesso de execução da tarefa; (2) tempo para completar a tarefa; (3) número de erros e frequência de erros; (4) ação apropriada após as mensagens de erro geradas pela aplicação; (5) número de tentativas de execução dos passos na tarefa.
