# Supplementary material for: Towards person-centered pain management in dementia: usability of a digital medical device in Portuguese residential care facilities
Source: Front Health Serv. 2026 Jun 24;6:1829082. doi: 10.3389/frhs.2026.1829082 (PMC13342171; doi:10.3389/frhs.2026.1829082)
Supplement: Supplementary file 3 [file Datasheet3.pdf]

Think-aloud script

<u>Sample</u>: Formal caregivers

Task 1 – <u>Log in to the application</u>:

**Preconditions**	• Smartphone with the PainChek app installed;<br>• Create a login (fictitious user);<br>○ Username: margarida.[redacted]<br>○ Password: ERPI#1teste<br>• Have the login information printed out;<br>• Have access to the Internet.
**Prompts**	Today, you are playing the role of Maria Ferreira, a professional at a residential facility for the elderly. You do not yet have a login for the PainChek app. Can you create a login for the app?<br>***Provide login details for the app***
**Assessed activity**	Log in to the PainChek mobile application using a specific user account:<br>1. Enter the fictitious username<br>2. Select *Next*<br>3. Enter the password<br>4. Select *Log In*
**Outcome measures**	(1) Task completion success rate; (2) time to complete the task; (3) number and frequency of errors; (4) appropriate action after error messages generated by the application; (5) number of attempts to perform the task steps.

Task 2 – Register a new resident in the application:

**Preconditions**	• The application must have some residents already registered: residents in general; "my residents"; some of these residents have assessments and others do not;<br>• Have the resident´s information printed: Maria José Santos, born July 5, 1951, female, resident at Lado do Lago, Dementia Ward, Room 3. **(CHANGE THE NAME FOR EACH TEST)**
**Prompts**	This is Maria José Santos **(NAME CHANGED FOR EACH TEST)**, a new resident. Maria is not yet registered in the PainChek application. Can you register her in the application and let me know when you are done? Here is the resident´s information.<br>***Present the participant with a printed document containing the resident´s information.***
**Assessed activity**	Users are asked to add a resident by following all the steps included in this process:<br>1. Log in to the app (one of the following options):<br>→ Option 1: Select *Add new resident*<br>→ Option 2: Select *My residents* and then select *Add new*<br>→ Option 3: Select *Residents* and then select *Add new*.<br>2. Fill in all the fields on the form (First Name, Last Name, Gender, Date of Birth, Location and Name of Residence, and Room).<br>3. Select *Save*.<br>4. The new profile should be found in the control panel, in the "Residents" section.
**Outcome measures**	(1) Task completion success rate; (2) time to complete the task; (3) number and frequency of errors; (4) appropriate action after error messages generated by the application; (5) number of attempts to perform the task steps.

Task 3 – Assess the pain of an existing resident using PainChek, via video:

**Preconditions**	The interviewer takes on the role of an actor and is instructed to:<br>• Grab the shoulder;<br>• Squint and show teeth;<br>• Sigh and sob;<br>• Respond abruptly to questions, such as "leave me alone" or "I don´t know".
**Prompts**	A Maria José Santos **(NAME CHANGED FOR EACH TEST)** has been complaining of shoulder pain. As a professional, I would like to assess the level of pain using the PainChek app, in *Video* mode. Can you carry out this assessment and let me know when you have finished and saved it?
**Assessed activity**	1. Open Maria José Santos ´resident profile (choose one of the following options):<br>→ Option 1: Select *Residents*, search Maria José Santos in the list, select *Maria José Santos*<br>→ Option 2: Select *My residents*, search Maria José Santos in the list, select *Maria José Santos* (only available if the resident is assigned to the user by the participant in task 3)<br>2. Select *Assess pain*<br>3. Select one of the following options:<br>→ Option 1: Activate *At rest*<br>→ Option 2: Activate *Post-movement* should not be chosen because Maria is sitting<br>4. Select *Next*<br>5. Activate *Video*<br>6. Point the smartphone camera at the resident´s face<br>7. Wait for the screen to become active<br>8. Select *Start analysis*<br>9. When finished, swipe left

	10. Complete the remaining 5 domain checklists<br>11. Select *View summary*<br>12. Select *Save*<br>13. Select *Save* in the pop-up window
**Outcome measures**	(1) Task completion success; (2) time to complete the task; (3) number and frequency of errors; (4) appropriate action after error messages generated by the application; (5) number of attempts to perform the task steps.

Task 4 – Review a previous pain assessment of a resident:

**Preconditions**	• Carlos Prata is available in the application;<br>• Carlos Prata´s profile must contain a previous assessment with the following data:<br>o Behavior domain, recorded as "Aggressive" and "Confused"<br>• Carlos Prata must be assigned to the user´s list of residents.
**Prompts**	One of your colleagues is asking about you resident Carlos Prata, as they are concerned about his recent behavior.<br>Could you please open the resident´s latest assessment and describe Carlos Prata´s behavior to update your colleague?
**Assessed activity**	1. Open the profile of resident Carlos Prata (choose one of the following options):<br>→ Option 1: Select *Residents*, search Carlos Prata in the list, Select Carlos Prata<br>→ Opção 2: Select *My residents*, search Carlos Prata in the list, Select Carlos Prata<br>2. Select the latest assessment<br>3. Select *Behavior* domain checklist<br>4. Tell the interviewer that the resident was aggressive and confused

**Outcome measures**	(1) Task completion success rate; (2) time to complete the task; (3) number and frequency of errors; (4) number of attempts to perform the task steps.

Task 5 – Interrupted assessment of an existing resident: delay in saving an assessment:

**Preconditions**	• António Dias is available on the application;<br>• António Dias is assigned to the user´s list of residents;<br>• The interviewer assumes the role of actor and is instructed to:<br>→ Keep their hand over their face, covering one eye, to prevent facial recognition;<br>→ Vocalize that they are in pain: "Oh, my head!" or something similar.
**Prompts**	This is resident António Dias, who recently fell and suffered a minor head injury and wants to have his pain assessed. Can you proceed with this assessment and let me know when you are finished?<br>*Interrupt the participant when they are completing the Movement domain, and continue with the Prompt*<br>You need to respond to an emergency at the end of the hallway, but you want to continue the pain assessment later. Would you like to postpone the assessment so you can return to it later? Can you do that and let me know when you´re done?
**Assessed activity**	1. Open the profile of resident António Dias (choose one of the following options):<br>→ Option 1: Select *Residents*, scroll to or search for António Dias in the list, Select *António Dias*<br>→ Option 2: Select *My residents*, scroll to or search for António Dias in the list, Select *António Dias*<br>2. Select *Assess pain*

	3. Select one of the following options:<br>→ Option 1: Activate *At rest*<br>→ Option 2: Activate *Post-movement*<br><br>4. Select one of the following options:<br>→ Option 1: Activate *Manual*<br>→ Option 2: Activate *Video*, then switch to *Manual* if video analysis does not work<br><br>5. Continue with the checklists (Voice and Movement domains)<br><br>***INTERRUPÇÃO***<br>6. Swipe to the last page (Body domain checklist)<br>7. Select *Show summary*<br>8. Select *Later*<br>9. Select *Later* in the pop-up window
**Outcome measures**	(1) Task completion success rate; (2) time to complete the task; (3) number and frequency of errors; (4) appropriate action after error messages generated by the application; (5) number of attempts to perform the task steps.

Task 6 – Complete a previously unfinished assessment:

**Preconditions**	Based on the previous scenarios: complete the incomplete assessment of resident António Dias.
**Prompts**	Resume the pain assessment of resident António Dias, with the aim of completing the assessment.
**Assessed activity**	1. Open the profile of resident António Dias (choose one of the following options):

	→ Option 1: Select *Residents*, scroll to or search for António Dias in the list, Select *António Dias*<br>→ Option 2: Select *My residents*, scroll to or search for António Dias in the list, Select *António Dias*<br><br>2. Select one of the following options:<br>→ Option 1: Select the orange assessment (not completed)<br>→ Option 2: Select *Assess pain*, then select *Update*<br><br>3. Select one of the incomplete domains to return to the associated checklist<br>4. Complete the remaining domains (Behavior, Activity, Body)<br>5. Select *View summary*<br>6. Select *Save*<br>7. Select *Save* in the pop-up window
**Outcome measures**	(1) Task completion success rate; (2) time to complete the task; (3) number and frequency of errors; (4) appropriate action after error messages generated by the application; (5) number of attempts to perform the task steps.

Task 7 – Archive a residente in the application:

**Preconditions**	• Ana Tomé is available as an active resident in the application;<br>• Ana Tomé is assigned to the user´s list of residents.
**Prompts**	Resident Ana Tomé is moving to another facility. Could you please remove her from the list of residents and let me know when you are done?
**Assessed activity**	1. Open the profile of resident Ana Tomé (choose one of the following options):<br>→ Option 1: Select *Residents*, scroll to or search for Ana Tomé in the list, Select Ana Tomé

	→ Option 2: Select *My residents*, scroll to or search for Ana Tomé in the list, Select *Ana Tomé*<br>2. Select ⋮<br>3. Select *File*
**Outcome measures**	(1) Task completion success rate; (2) time to complete the task; (3) number and frequency of errors; (4) appropriate action after error messages generated by the application; (5) number of attempts to perform the task steps.

Task 8 – <u>Restore an archived resident in the application</u>:

**Preconditions**	Based on the previous scenario, we want to restore the file for resident Ana Tomé.
**Prompts**	Resident Ana Tomé is returning to the Lado do Lago residence. Can you restore her profile to the list of residents?
**Assessed activity**	1. Select one of the following options:<br>→ Option 1: Select *Residents*<br>→ Option 2: Select *My residents*<br><br>2. Select *File*<br>3. Select ⋮ to the right of resident Ana Tomé´s profile in the list<br>4. Select *Restore*<br>5. Select *Yes*<br>6. *Select <Home>* to return to the home page
**Outcome measures**	(1) Task completion success rate; (2) time to complete the task; (3) number and frequency of errors; (4) appropriate action after error messages generated by the application; (5) number of attempts to perform the task steps.

Task 9 – Log out:

**Preconditions**	Based on the previous scenario, the user wants to exit the application.
**Prompts**	When finished: Can you log out and close the application?
**Assessed activity**	1. Open the Menu by selecting the icon ≡<br>2. Select *Log Out* or *Exit*
**Outcome measures**	(1) Task completion success rate; (2) time to complete the task; (3) number and frequency of errors; (4) appropriate action after error messages generated by the application; (5) number of attempts to perform the task steps.
