# Supplementary material for: Towards person-centered pain management in dementia: usability of a digital medical device in Portuguese residential care facilities
Source: Front Health Serv. 2026 Jun 24;6:1829082. doi: 10.3389/frhs.2026.1829082 (PMC13342171; doi:10.3389/frhs.2026.1829082)
Supplement: Supplementary file 4 [file Datasheet4.pdf]

Questionário Sociodemográfico e PSSUQ-PT

Dados de identificação:

1. Código de participante *(código fornecido pela candidata)*: __________
2. Data da recolha de dados: __________

Secção I – Dados Sociodemográficos

3. Selecione a sua profissão:
   ( ) Enfermeiro(a)
   ( ) Médico(a)
   ( ) Psicólogo(a)
   ( ) Fisioterapeuta
   ( ) Assistente operacional
   ( ) Assistente social
   ( ) Terapeuta ocupacional
   ( ) Outra: __________

4. Há quanto tempo trabalha numa Estrutura Residencial para Pessoas Idosas?
   (Por favor, indique a sua resposta em anos) __________

5. Qual é a sua idade? __________

6. Qual é o seu género?
   ( ) Feminino
   ( ) Masculino

7. Qual é o seu nível de escolaridade?
   ( ) 1º e 2º ciclos do ensino básico (até ao 6º ano)
   ( ) 3º ciclo do ensino básico (até ao 9º ano)
   ( ) Ensino secundário (10º a 12º ano)
   ( ) Bacharelato ou licenciatura ou nível equivalente
   ( ) Mestrado ou nível equivalente
   ( ) Doutoramento

Secção II – Questionário de usabilidade (PSSUQ-PT)

Após experimentar e analisar a aplicação PainChek, por favor, selecione o seu nível de concordância com as afirmações seguintes, tendo em conta a seguinte escala de Likert.

	1	2	3	4	5	6	7	
Concordo Totalmente	○	○	○	○	○	○	○	Discordo Totalmente

1U. Em geral, estou satisfeito com a facilidade de utilização deste sistema.

2U. Este sistema foi simples de utilizar.

3U. Consegui completar as tarefas e os cenários utilizando este sistema.

4U. Consegui completar rapidamente as tarefas e cenários utilizando este sistema.

5U. Consegui completar as tarefas e os cenários com eficiência utilizando este sistema.

6U. Senti-me confortável a utilizar este sistema.

7U. Foi fácil aprender a utilizar este sistema.

8U. Acredito que me tornaria rapidamente produtivo se utilizasse este sistema.

9U. O sistema deu mensagens de erros que me indicaram claramente como resolver os problemas.

10U. Sempre que cometi um erro durante a utilização do sistema, consegui recuperar de forma fácil e rápida.

11U. A informação fornecida pelo sistema (como ajuda online, mensagens no ecrã ou outra documentação) foi clara.

12U. Foi fácil encontrar a informação que precisava.

13U. A informação fornecida pelo sistema foi fácil de entender.

14U. A informação foi eficaz para me ajudar a completar as tarefas e os cenários.

15U. A organização da informação que o sistema transmitiu foi clara.

16U. A interface do sistema foi agradável.

17U. Gostei de utilizar a interface deste sistema.

18U. Este sistema tem todas as funcionalidades e capacidades que eu esperava.

19U. Em geral, estou satisfeito com este sistema.

Secção III – Questões abertas adicionais

1. O que mais gostou na aplicação PainChek? ____
2. O que menos gostou na aplicação PainChek? ____
3. Que melhorias sugere? ____
